# Effects of a 6-Week Internet-Based Stress Management Program on Perceived Stress, Subjective Coping Skills, and Sleep Quality

**DOI:** 10.3389/fpsyt.2020.00463

**Published:** 2020-05-25

**Authors:** Tobias Stächele, Gregor Domes, Magdalena Wekenborg, Marlene Penz, Clemens Kirschbaum, Markus Heinrichs

**Affiliations:** ^1^Laboratory for Biological and Personality Psychology, Department of Psychology, University of Freiburg, Freiburg, Germany; ^2^Outpatient Psychotherapy Clinic and Research Unit for Stress-Related Disorders, University of Freiburg, Freiburg, Germany; ^3^Department of Biological and Clinical Psychology, University of Trier, Trier, Germany; ^4^Department of Biological Psychology, Technical University of Dresden, Dresden, Germany

**Keywords:** Internet-based stress management training, prevention, health, evaluation, perceived stress, coping, sleep

## Abstract

Occupational stress management intervention programs are known to be effective in preventing stress-related health burden. Two essential mechanisms underlie this effect: (i) a reduction in perceived stress (e.g., *via* relaxation-oriented techniques), and (ii) an improvement in coping skills (e.g., *via* cognitive-behavioral interventions). While relaxation-oriented interventions are more frequently employed in occupational settings, cognitive-behavioral stress management interventions reveal stronger effects on stress-related outcomes. As an effective and economic strategy, the dissemination of stress management programs *via* the internet is soaring, but most internet-based programs focus on relaxation or reducing perceived stress. In the present study, we examined the effects of a self-guided, cognitive-behavioral 6-week Internet-Based Stress Management (IBSM) program on perceived stress, coping skills, emotional exhaustion, depressive symptoms, and sleep quality. The IBSM consists of six modules focusing on the improvement of stress management skills such as dealing with acute stress, building up resources, or reappraising stress-facilitating cognitions. The participants have to work through the content on a weekly basis, requiring about 30 min per week followed by a transfer task lasting another 30 min. Healthy employees reporting elevated stress were recruited over the Internet and then randomly assigned to the IBSM group or a waiting list control group. A total of 134 participants completed all assessments before and after the training or waiting period. The IBSM group reported lower subjective stress levels after the program than the control group. In addition, the IBSM group exhibited improved coping skills and better sleep quality. Emotional exhaustion was reduced in both groups, while depressive symptoms did not change. These results suggest that a brief, 6-week, cognitive-behavioral internet-based stress management program improves coping skills, sleep quality, and well-being, and reduces the perceived stress of employees. Our results might encourage large-scale studies on the long-term stability and clinical efficacy of internet-based programs. The trial is registered in the German Clinical Trials Register (DRKS00014837) URL. https://www.drks.de/drks_web/navigate.do?navigationId=trial.HTML&TRIAL_ID=DRKS00014837

## Introduction

Occupational stress is a widespread phenomenon. In Germany, more than 60% of all employees feel frequently or sometimes stressed (1). Chronic occupational and work-related stress are well-known risk factors for various chronic health burdens (2), psychiatric disorders [e.g., depression (3)], and poor sleep quality (4–6). The overall costs of work-related stress are estimated to exceed 20 billion Euro in Europe and 180 billion US$ annually (7).

Psychological interventions to teach effective ways of handling a heavy workload by improving individual stress management skills have been proposed as a promising means of preventing the health burden caused by work-related stress (8).

There is solid evidence that interventions based on cognitive-behavioral therapy provide particular support for persons suffering from work-stress (9, 10). Cognitive-behavioral therapy is a set of interventions sharing the core assumption that maladaptive cognitive processes are a main factor in perpetuating emotional distress and behavioral problems (11). This therapy approach helps potential participants become active partakers during treatment, being encouraged to develop and test rectified and functional cognitions, emotions, and behavioral patterns. Transferred to the field of work-related stress, these core assumptions remain the same (12). Stress-management training interventions for persons suffering from work-stress consist of a combination of behavioral and cognitive components [e.g., (12, 13)]. The behavioral part aims to develop skills such as assertiveness, relaxation, problem solving or time management. The cognitive part follows a transactional model of stress (14) and includes techniques to modify stress-related cognitions and coping appraisals [e.g., (15)]. Gardner et al. (16) showed that the changes in cognitive appraisals seem to drive an intervention's effect.

In healthy but stressed individuals, such interventions are directed to (i) reduce perceived stress and (ii) improve individual coping skills. Whereas some programs focus on reducing stress levels by fostering relaxation, recovery, and recreation [e.g., *via* relaxation techniques (17)], others focus on enhancing individual coping skills by improving abilities to effectively deal with stressful events, change the perception and appraisal of situations, and modify the emotional meaning of stressors (e.g., *via* applying cognitive-behavioral techniques). Concerning face-to-face stress management training programs in an occupational setting representing a wide range of different occupations, the latest meta-analysis of Richardson & Rothenstein (10) included 55 studies with about 2,800 participants and found an overall stress-reducing effect of stress management training programs of *d* = 0.52 (95% CI, 0.36–0.69). The effects differ according to the training content. Notably, the strongest impact on perceived stress was identified in cognitive-behavioral-oriented programs (*d* = 1.16; 95% CI, 0.46–1.87), outperforming relaxation oriented training (*d* = 0.50; 95% CI, 0.31–0.69), and multimodal programs (*d* = 0.24; 95% CI, 0.09–0.39).

Nowadays, online-based interventions offer a welcome and growing opportunity to offer stress management training to individuals. The general transfer of psychological interventions from face-to-face to structured self-help online training programs relies mainly on cognitive-behavioral therapy principles (18). The guidance of the therapy process by a health professional differs between applications—from no contact at all up to several contacts per week. Baumeister et al. (19) found a minor advantage of providing guidance concerning the therapy outcomes, discussing that to prevent mental disorders caused by economic factors, unguided programs might play an important role. In a meta-analysis, Andersson et al. (20) reported equal treatment success following face-to-face versus internet based psychological treatments in conjunction with various mental disorders. Nevertheless, a direct comparison of stress management training interventions that solely differ in a computer based and a person-centered presentation revealed no clear results (21–23).

In particular, self-help interventions that are Internet-based and standardized reveal several advantages over face-to-face training, including flexible and time-independent availability using multimedia content of consistent quality (24, 25). On the other hand, the main disadvantages are the high attrition and higher drop-out rates. A potential limitation of internet-based interventions is an imprecise diagnosis (18).

Since the study by Zetterquist et al. (26), several standardized, internet-based, stress management training programs have been developed and evaluated. Some focus on different occupations [e.g., (27, 28)] or college students [e.g., (29)], while others are targeted at stress-related health complaints (30) or chronic health burden (31, 32). Heber et al. conducted a meta-analysis including web- and mobile-based stress management training programs and detected medium effects on perceived stress (*d* = 0.43; 95% CI, 0.31–0.54) including 26 studies with about 4,200 participants (33). Another finding was an effect of the program's duration. Summarizing these results, in line with face-to-face training interventions, internet-based programs lasting 5 to 8 weeks demonstrate the highest effectiveness, whereas programs lasting longer revealed no additional effect.

The effects of online interventions on improving individual coping skills in healthy individuals have been slightly explored to date. Hasson et al. (28) investigated the effects of a web-based, stress management training intervention lasting six months in employees at different companies by using visual analog scales and found a significantly stronger improvement in coping skills in their training group compared to their waiting group. In an previous study from our laboratory evaluating the psychobiological effects of a cognitive-behavioral, internet-based training program, we demonstrated that modifying appraisals by a cognitive-behavioral training intervention yielded similar effects on the reducing perceived stress as did a relaxation-focused online training intervention, but the cognitive-behavioral training intervention outperformed the mere relaxation-based one in terms of the psychobiological stress-response to an acute laboratory stressor (34).

Elevated stress and impaired sleep are strongly associated (4) and cognitive-behavioral therapy is known to have positive effects (35). A specific internet-based therapy on a cognitive-behavioral basis has shown significant effects alleviating poor sleep and insomnia, (36). Ebert et al. (30) demonstrated a strong effect reducing insomnia severity (*d* = 1.37) with an internet-based training intervention specifically developed to restore depleted resources and alleviate sleeping problems. Concerning sleeping quality there is a difference in effects between online training interventions with or without guidance. Self-help programs reveal smaller effects than guided programs; on the other hand, there is an economic advantage to distributing standardized and effective interventions to people who are hard to reach. Ebert et al. (37) showed small to medium effects (*d* = 0.33) of an unspecific self-guides stress management training program on sleep quality.

Further secondary outcomes were examined in several studies evaluating cognitive-behavioral stress-management interventions in healthy individuals delivered face-to-face or internet-based, including emotional exhaustion [e.g., (12, 38, 39)] or life satisfaction [e.g., (40, 41)]. For an overview of web-based interventions, see a recent review by Ryan et al. (42).

In the present study, we investigated effects of a standardized self-guided cognitive-behavioral 6-week ISBM program distributed solely over the internet, without any personal contact with a mental health professional on healthy employees. Therefore, we compared a training-group to a waiting-list control group. We hypothesized that the training group would improve their stress management skills and perceive reduced stress, which in turn would lead to better sleeping quality, and thus reduce the subsequent health burden.

## Methods

### Study Design

A randomized controlled trial was conducted with two conditions: a standardized self-guided IBSM program and a waiting condition. Participants randomized to the waiting group were allowed to access the training program after the post-treatment measure without further professional support. Assessments took place at baseline (T1) and post-treatment (8 weeks, T2). The study protocol was approved by the institutional review board of the University of Freiburg (Germany). The trial is registered in the German Clinical Trials Register (DRKS00014837).

### Participants

We employed two ways to recruit participants. Potential healthy participants who enrolled for a larger project announced as longitudinal study on stress and burnout in the region of Dresden (Germany) (8), were contacted by e-mail and informed about the possibility of participating in an evaluation study of a novel IBSM program. Participants were also recruited *via* newspapers advertisements. In all, 432 persons responded to our invitation and were sent a link to an online screening test to determine eligibility in line with our inclusion and exclusion criteria. We included individuals who claimed to be currently working full-time or at least half-time, were above the age of 18 years, experiencing elevated stress levels and interested in improving their stress management skills, and who had adequate internet access and sufficient skills in reading and writing German. We excluded individuals who were currently under or who had within the previous 6 months undergone medical or psychotherapeutic treatment for a psychiatric disorder or a mental-health problem, and any who reported traumatic experiences or suicidal ideation within the previous 6 months. All information concerning our participants' inclusion or exclusion criteria were collected *via* online questionnaires.

**Figure 1:**
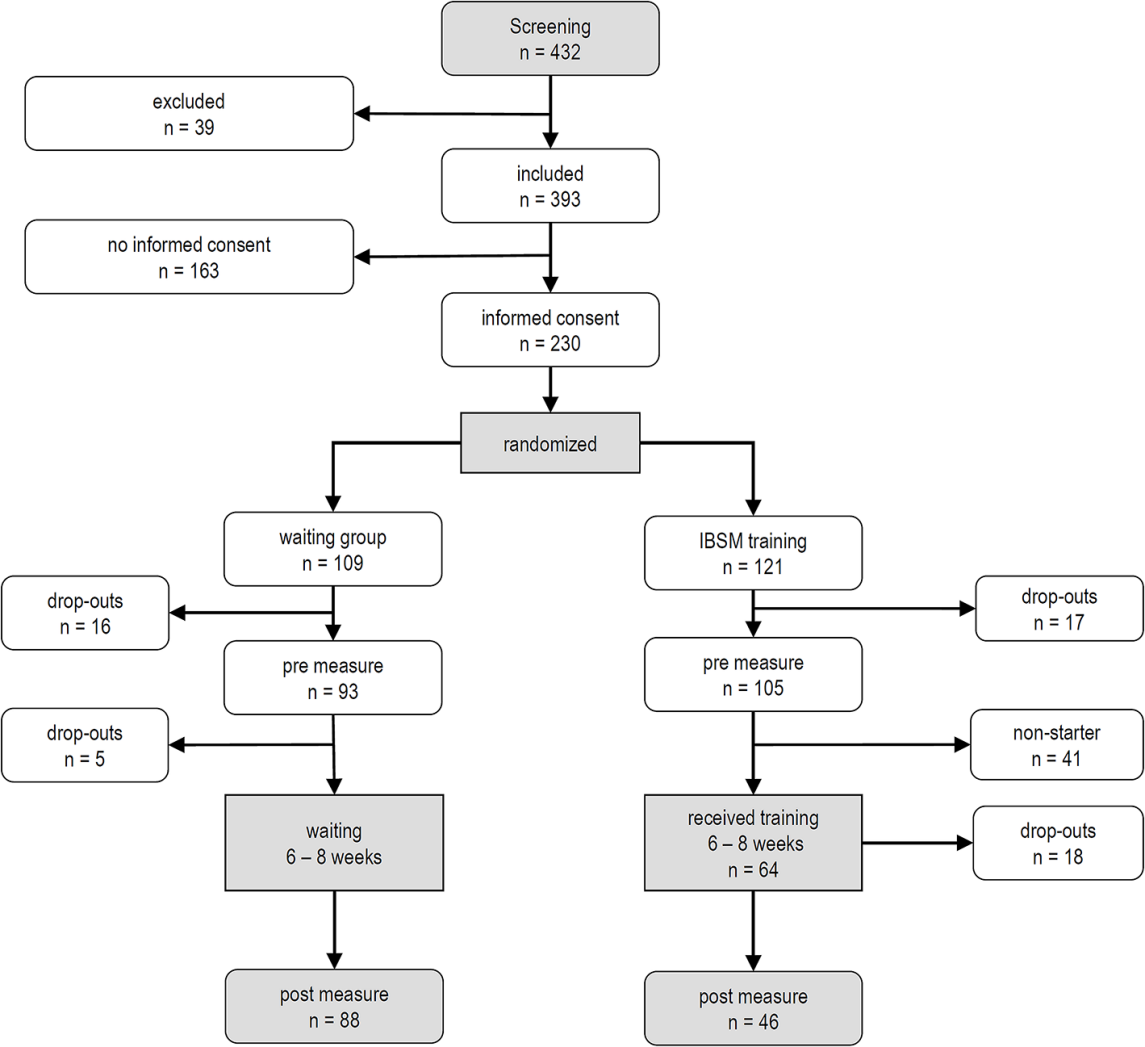
Flow of participants. IBSM, Internet-Based Stress Management Program.

In total, 393 passed our screening procedure and were sent written informed consents; 230 returned the consent form and were randomized by an individual random assignment (“coin toss”) to one of our two treatment conditions: the IBSM program or the waiting list control group. Pre-treatment questionnaires (t1) were completed by 105 participants in the IBSM group, and 93 in the control group. After 8 weeks, 46 participants in the IBSM group and 88 in the control group completed the post-treatment questionnaires (t2) and were available for statistical analyses. For the flow of participants, see **Figure 1**.

### The Internet-Based Stress Management Program

The Internet-Based Stress Management (IBSM) program is a self-guided cognitive-behavioral training program, structured in six modules. The program addresses both cognitive processes to improve adequate reappraisals of demanding situations, and behavioral strategies for managing resources more beneficially.

Hence, the evaluated ISBM program relies on a transactional stress-model by Lazarus (14, 43), using interventions whose success has been proven [e.g., (26, 44)]. The aim is to improve coping skills for acute stressful events and to facilitate coping strategies for successful re-appraisals of demanding work-related situations. The participants are, therefore, asked to define and work on their own stressful situations to transfer and test the presented behavioral pattern and stress-related appraisals.

After receiving an anonymous access code to start training, participants could register in the web-based training program and work on the lessons on a weekly basis. During a 6-week training period, each participant is requested to complete one module per week. As the training is available with no time limit, the participants are free to decide, when during the week they will execute the content. The subsequent module is unlocked each week, provided the previous module has been completed successfully. To finish a module, participants had to work through the module as well as elaborate on the content by transferring it to an individual, work-related, daily stress-situation. Therefore, after executing the content, each module ends with the request “Now it´s your turn” and the participants have to work on their own stressful situation, adapting the skills presented to alleviate their stress. If the participants fail to reach their weekly goal, they receive a standardized e-mail reminder sent automatically.

The six weekly modules contained the following: (1) understanding stress and the effects of stress on health and performance, (2) strategies to deal with acute stress and a first-aid kid, (3) creating personal space and building up resources against stress, (4) reappraising stress-facilitating cognitions, (5) active coping strategies and systematic problem-solving, and (6) keeping in balance and transfer to everyday life (**Table 1**). The IBSM program was available *via* the internet and could be accessed with a standard PC, tablet or mobile phone. The content was visualized through various web-based multimedia applications, e.g., text, video clips, audio files, animations, and interactive elements. Total working time for a single module was approx. 45 min. For a detailed description of the modules included in the IBSM program, see Domes et al. (34).

**Table 1:** Modules and content of the IBSM program.

Module	Content
1. Understanding stress	–Psychobiological basics of stress and related consequences –Becoming familiar with key effective means of managing stress
2. First aid	–Short term strategies to deal with acute stress –Design and use a personal first-aid program in everyday life
3. Creating personal space	–Effective time management –Building resources and reinforcing recreational competencies
4. Rethinking	–Reduction of stress-promoting cognitions –Rethinking judgmental tendencies encouraging stress
5. Active coping	–Systematic problem solving –Solution-oriented coping activities during stress
6. Keep your balance	–Integration and transfer to everyday life –Euthymic elements and self-care

### Outcome Measures

#### Primary Outcome Variables

As a global measure of perceived stress, we used the 10-item version of the Perceived Stress Scale, PSS (45). The PSS demonstrated good reliability (Cronbach alpha = .84–.86) (45, 46) and has been used as a sensitive measure to track perceived stress in longitudinal studies [e.g., (34, 47, 48)]. The validation of the German translation (N = 2,463) of the PSS-10 has shown, in line with previous research, strong positive correlations with depression, anxiety, fatigue, and negative correlations with life satisfaction (46). For the present study with a 6-week training period, participants rated the items in reference to the previous two weeks, as that timeframe is valid to represent a potential change due to the intervention.

To assess stress coping abilities, we used a version of the Measure of Current Status (MOCS) originally developed by Carver and colleagues (49), namely the adapted German version of the MOCS, the ISBF (“Inventar zur Erfassung von Stressbewältigungsfertigkeiten” English translation: Inventory to Assess Stress Management Skills) already validated as a brief measure for self-reported coping skills with good psychometric indices (e.g., Cronbach alpha = .83). High levels on the ISBF are associated with reduced psychobiological reactivity to acute psychosocial stress induced with a laboratory stressor (50). The ISBF is a 14-item questionnaire enabling the assessment of the subjective overall effectiveness of stress coping and of coping skills in different domains: (1) cognitive strategies and problem solving, (2) of the identification and use of social resources, (3) of relaxation abilities, (4) of adequate anger expression and assertiveness, (5) and the perception of bodily tension.

#### Secondary Outcomes

In addition to specific measures of stress and coping, life satisfaction was assessed *via* the Satisfaction with Life Scale (SWLS). The German version of the SWLS was evaluated in a large German sample (*N* = 2,519) and revealed very good reliability (e.g., Cronbach alpha = .92) and exhibited good convergent validity, e.g., by correlations with depression (r = −.44) and social support (r = .39) (51).

To assess sleep quality, we employed the Pittsburg Sleep Quality Index (PSQI) and calculated the PSQI total score as a global measure of overall sleep quality for the previous 2 weeks for the purpose of this study. The PSQI demonstrated acceptable reliability of Cronbach´s alpha = .75 in a widespread study in the general population (N = 9,285) (52) and has been widely used as a sensitive and valid measure of sleep quality (52–54).

Finally, we included the emotional exhaustion subscale of the Maslow Burnout Inventory, MBI-EE (55). Previous studies have evaluated the MBI-EE subscale as a measure of emotional exhaustion in work-related contexts with good reliability and a Cronbach's alpha exceeding. 84 [e.g., (56, 57)]. We approximated the severity of depressive symptoms *via* the nine-item Patient Health Questionnaire; PHQ-9 (58). The PHQ-9 has been validated in a representative German sample (N = 2,066) revealing good internal consistency of Cronbach´s alpha = .89 (49).

### Statistical Analysis

Effects of time and group on primary and secondary outcome variables were tested with separate two-way mixed repeated measures ANOVAs as completers-only analyses. For stress-coping abilities, we explored which subscales appeared as the main factors calculating subsequent ANOVAs for the subscales. Effect-sizes were calculated as partial eta-squared. Pre-post effect sizes are given as Cohen's d. All statistical analyses were done with SPSS for Windows (Version 24). Level of significance was set to *p* < .05.

## Results

### Participant Characteristics

Randomized groups did not differ in age, sex distribution, occupational background, and pre-stress level. However, the IBSM training group reported a higher level of workload in terms of working hours per week (see **Table 2**).

**Table 2:** Group characteristics.

	IBSM, n = 46			Controls, n = 88		
Age (m/s.d.)	44.4 (9.8)			43.8 (10.6)		t = 0.31; *p* = 0.761
Sex (m/f)	16/30	35%/65%		24/64	27%/73%	χ^2^ = 0.81; *p* = 0.428
Employed (n)	35	76%		74	84%	χ^2^ = 1.21; *p* = 0.258
Working hours per week (m/s.d.)	39.7 (10.8)			44.1 (7.8)		t = 2.74; *p* = 0.007
Perceived stress level (m/s.d.)	21.4 (5.3)			21.0 (5.7)		t = 0.351; *p* = 0.726

The participants represented a wide range of occupations including office and administration Jobs (35.8%), engineers and technical staff (24.6%), health professionals (20.9%), social occupations (9%), teachers (6.7%), and others (3%).

### Primary Outcome Variables

#### Stress Level

**Figure 2:**
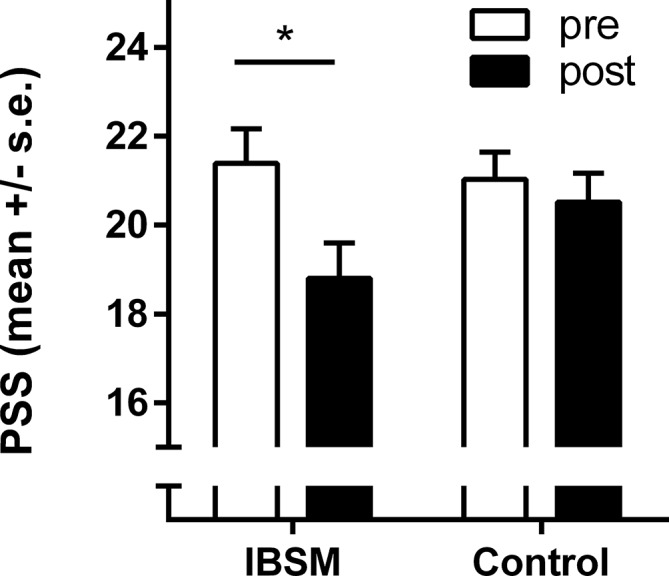
Pre-post differences in the Perceived Stress Scale (PSS) for the Internet-Based Stress Management (IBSM) group and the control group. Bars represent mean ± s.e.; *p < 0.05.

In the PSS, we detected a significant effect of time (*F*[1,132] = 12.6; *p* = .001; η^2^ = .09) and a significant time x group interaction (*F*[1,132] = 5.46; *p* = .019; η^2^ = .04), indicating an overall reduction in the reported stress level that was more pronounced in the IBSM group. In the IBSM group, we noted a medium pre-post effect size of Cohen's *d* = 0.49 (t[45] = 3.31, *p* = .002), whereas no such effect was evident in the control group, Cohen's *d* = 0.12 (t[87] = 1.06, *p* = .292) (see **Figure 2**).

#### Stress Coping Abilities

**Figure 3:**
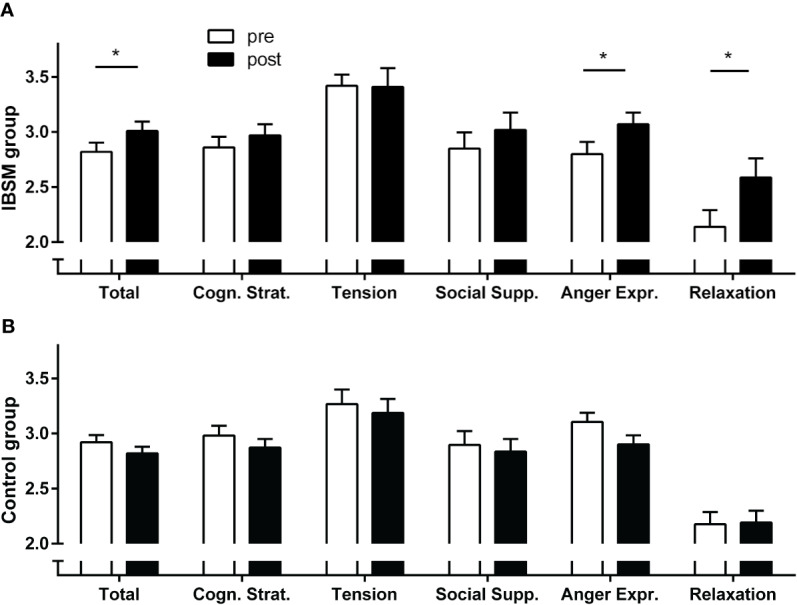
Pre-post training stress coping abilities measured with the ISBF **(A)** in the Internet-Based Stress Management (IBSM) group and **(B)** in the control group. Bars represent mean ± s.e.; *p < 0.05.

For stress-coping skills (total score), a significant group-by-time interaction indicated an overall increase in reported stress-coping skills in the IBSM group compared to the control group (*F*[1,132] = 11.46; *p* = .001; η^2^ = .08).). For the total score, the IBSM group attained a medium pre-post effect size of Cohen's *d* = 0.39 (t[45] = 2.464, *p* = .011), whereas, as the control group reported lower coping-skills levels after the waiting period, Cohen's *d* = −0.22 (t[87] = −2.06, *p* = .042). For the subscales, we found increased abilities in appropriate anger expression (*F*[1,132] = 18.14; *p* < .001; η^2^ = .12) and relaxation (*F*[1,132] = 11.30; *p* = .001; η^2^ = .08) (see **Figure 3**).

### Secondary Outcome Measures

**Figure 4:**
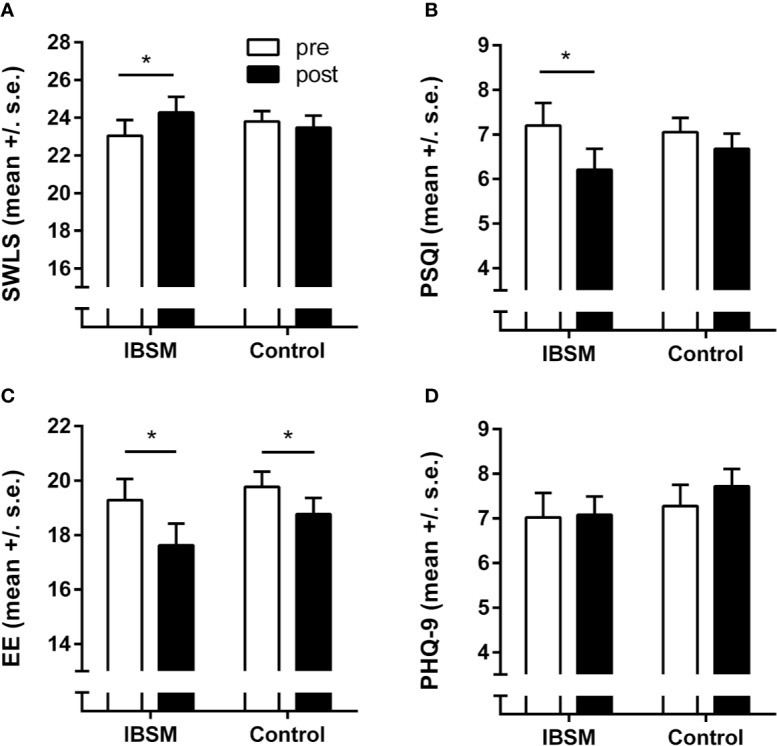
Secondary outcome variables. Effects of the Internet-Based Stress Management (IBSM) program on **(A)** life satisfaction (SWLS), **(B)** sleep quality (PSQI), **(C)** emotional exhaustion (MBI-EE), and **(D)** depressive symptoms (PHQ-9). Bars represent mean ± s.e.; *p < 0.05.

Life satisfaction as measured with the SWLS displayed a trend towards a differential effect in both groups (*F*[1,132] = 3.66; *p* = .058; η^2^ = .03): Life satisfaction significantly increased in the IBSM group (t[45] = −2.053; *p* = .046; Cohen's *d* = 0.30), whereas there was no such effect in the control group (t[87] = 0.657; *p* = .513; Cohen's *d* = 0.07) (see **Figure 4A**). We observed a similar pattern for sleep quality: Whereas the IBSM group reported an increase in sleep quality over the course of the stress management training program (t[45] = 3.04; *p* = .004; Cohen's *d* = 0.34), the control group showed no such effect (t[87] = 1.43; *p* = .157; Cohen's *d* = 0.16), although in the ANOVA the group-by-time interaction was not significant (*F*[1,132] = 2.12; *p* = .15; η^2^ = .02) (see **Figure 4B**).

However, we identified no differential effects for emotional exhaustion as a key facet of burnout and depressive symptoms. Emotional exhaustion revealed a decline in the course of the experiment in both groups (main effect of time: *F*[1,132] = 15.0; *p* < .001; η^2^ = .10) (see **Figure 4C**). Depressive symptoms remained unaltered during the experiment in both groups (all *F* < 2.0; *p* > .15) (see **Figure 4D**).

## Discussion

The goal of this study was to investigate effects of a 6-week self-guided cognitive-behavioral Internet-Based Stress Management (IBSM) program. We, therefore, conducted a randomized controlled trial comparing an IBSM group with a waiting control group, and analyzed the effects on perceived stress and subjective coping skills as primary outcome measures. In addition, we assessed sleep quality, depressive symptoms, burnout, and life satisfaction as secondary outcome measures.

Our results show that a 6-week self-guided IBSM program reduced self-reported perceived stress levels in the training group compared to the waiting list control group, thus replicating the previously found stress-reducing effects in a larger sample of participants (34). Moreover, the IBSM-group participants improved their stress-coping abilities compared to the waiting group. For secondary outcomes, we detected an increase in sleep quality and improved life satisfaction in the IBSM group. The effects on emotional exhaustion and depressive symptoms did not differ between groups: while emotional exhaustion declined in both groups, depressive symptoms did not change significantly in either group.

Hence, our results suggest that a self-guided online training program involving no contact with a health professional can support employees in handling everyday stress. Regarding perceived stress, our findings resemble the effect-sizes reported by Heber et al. (33) in their meta-analysis (*d* = 0.49). Although stress reduction might be regarded the most relevant outcome of a stress management program, the effects of such a training should not be limited to the perceived stress level, but rather focus much closer on improving coping abilities to enable demanding daily stressors to be better managed. Accordingly, we identified a small to medium effect (*d* = 0.39) in the training group, while the waiting group's self-reported coping abilities declined (*d* = −0.22).

Regarding our further outcomes, sleep quality is an important indicator for stress-related health complaints (5, 59). In several studies, different internet-based programs showed that a reduction in stress is accompanied by improved sleep quality (27, 37). The small to medium effects our study revealed (*d* = 0.34) are similar to other self-help stress management training programs not developed specifically to treat impaired sleep.

This study supports the finding that self-help internet-based stress management training programs are suitable to help healthy employees strengthen their ability to handle daily stressors better and ease stress-related complaints. Taking into account that the placebo effect might have affected our results, it is difficult to judge whether improvement occurred because of the treatment. Research indicates that some individuals who do not undergo treatment often improve, but those undergoing some type of psychotherapy are more likely to improve faster, and with a lower chance of relapsing (60). In this study, we assessed self-report measures exclusively. However, in a previous study from our laboratory evaluating this IBSM program (34) using a standardized laboratory stress protocol [TSST-G, (61)], we demonstrated that the training also exerts a positive impact on psychobiological stress parameters.

The training program we investigated consists of a variety of potent cognitive-behavioral stress management techniques. While the perceived stress level is a frequent outcome of studies evaluating the effects of stress management interventions, this study focuses beyond that on analyzing the improvement in coping skills in a structured way, using a validated psychometric instrument.

In this study, we focused on a low entry threshold to include a wide range of healthy people interested in improving their stress management skills. We investigated an employment of a standardized and scalable training program but with restricted personal contact limited only to technical problems. In our study, we noted a dropout rate of 28% of the participants who started the treatment, a percentage that is comparable to other internet-based stress management interventions for healthy employees [e.g., (27)] and well within the range of dropout rates reported in conjunction with internet-based psychological treatment programs [ranging from 2 to 83% with a 31% average (62)]. However, a limitation of the study is the unexpected number of pre-treatment dropouts (39%). We are aware of no other study showing similar numbers except an online intervention for social phobia (63). Perhaps the precise information we had provided on the program and the ultimate effort failed to meet our participants' expectations. In an earlier study (34) we showed an overall dropout rate of 18% and no pre-treatment dropouts. That study's main difference to this one was a single face-to-face contact with participants at study entry, which might have increased compliance and reduced the dropout rate. Finally, dropout rates were significantly higher in the IBSM than the control group, an effect we can attribute to the fact that the waiting group was promised access to the IBSM program after the second measurement. Thus, many control participants completed the post-measurement, while many IBSM participants were not motivated to complete the second measurement. We were, therefore, unable to collect data to estimate the stability of the effects reported here. To that end, future studies comparing different treatments (e.g., online vs. face-to-face) and incorporating follow-up measures are needed.

## Conclusion

In sum, the present study demonstrates that a brief 6-week cognitive-behavioral Internet-Based Stress Management program promotes coping skills, improves sleep quality and well-being, and reduces the perceived stress of healthy participants reporting moderate levels of work-related stress. Our results might encourage large-scale studies on the long-term stability and clinical significance of such beneficial effects of internet-based stress-management interventions. Altogether, the main strengths of such stress management training programs are their low entry threshold character, their cost-effectiveness, and potential reach to preventively improve stress-coping skills in working populations.

## Data Availability Statement

The datasets generated for this study are available on request to the corresponding author.

## Ethics Statement

The studies involving human participants were reviewed and approved by Institutional Review Board of the University of Freiburg. The participants provided their written informed consent to participate in this study.

## Author Contributions

TS, GD, and MH designed the study. GD, MW, and MP conducted data collection. GD and TS analyzed the data. All authors contributed to interpreting these results and to writing the manuscript. All authors have approved the final version of this article.

## Conflict of Interest

The authors declare that the research was conducted in the absence of any commercial or financial relationships that could be construed as a potential conflict of interest.

## References

[1] Entspann dich . Deutschland - TK-Stressstudie 2016. Available at: https://www.tk.de/resource/blob/2026630/9154e4c71766c410dc859916aa798217/tk-stressstudie-2016-data.pdf.

[2] Siegrist J Li J Work Stress and the Development of Chronic Diseases. Int J Environ Res Public Health (2018) 15:536. 10.3390/ijerph15030536 PMC5877081 29547566

[3] Cohen BE Edmondson D Kronish IM State of the Art Review: Depression, Stress, Anxiety, and Cardiovascular Disease. Am J Hypertens (2015) 28:1295–302. 10.1093/ajh/hpv047 PMC4612342 25911639

[4] Âkerstedt T Psychosocial stress and impaired sleep. Scand J Work Environ Health (2006) 32:493–501. 10.5271/sjweh.1054 17173205

[5] Âkerstedt T Knutsson A Westerholm P Theorell T Alfredsson L Kecklund G Sleep disturbances, work stress and work hours: a cross-sectional study. J Psychosom Res (2002) 53:741–8. 10.1016/S0022-3999(02)00333-1 12217447

[6] Hapke U Maske U Scheidt-Nave C Bode L Schlack R Busch MA Chronischer Stress bei Erwachsenen in Deutschland. Bundesgesundheitsb. (2013) 5:749–54. 10.1007/s00103-013-1690-9 23703494

[7] Hassard J Teoh KRH Visockaite G Dewe P Cox T The cost of work-related stress to society: A systematic review. J Occup Health Psychol (2018) 23:1–17. 10.1037/ocp0000069 28358567

[8] Penz M Wekenborg MK Pieper L Beesdo-Baum K Walther A Miller R The Dresden Burnout Study: Protocol of a prospective cohort study for the bio-psychological investigation of burnout. Int J Methods Psychiatr Res (2018) 27:e1613. 10.1002/mpr.1613 29611872 PMC6877172

[9] Van der Klink JJ Blonk RW Schene AH& van Dijk, FJ. The Benefits of Interventions for Work-Related Stress. Am J Public Health (2001) 91:270–6. 10.2105/AJPH.91.2.270 PMC1446543 11211637

[10] Richardson KM Rothstein HR Effects of occupational stress management intervention programs: A meta-analysis. J Occup Health Psychol (2008) 13:69–93. 10.1037/1076-8998.13.1.69 18211170

[11] Hofmann SG Asnaani A Vonk IJJ Sawyer AT Fang A The efficacy of cognitive behavioral therapy: a review of meta-analyses. Cognit Ther Res (2012) 36:427–40. 10.1007/s10608-012-9476-1 PMC3584580 23459093

[12] Querstret D Cropley M Kruger P Heron R Assessing the effect of a Cognitive Behaviour Therapy (CBT)-based workshop on work-related rumination, fatigue, and sleep. Eur J Work Organ Psychol (2016) 25:50–67. 10.1080/1359432X.2015.1015516

[13] De Vente W Kamphuis JH Emmelkamp PMG Blonk RWB Individual and group cognitive-behavioral treatment for work-related stress complaints and sickness absence: A randomized controlled trial. J Occup Health Psychol (2008) 13:214–31. 10.1037/1076-8998.13.3.214 18572993

[14] Lazarus RS Psychological stress and the coping process. New York, NY, US: McGraw-Hill (1966).

[15] Palmer S Gyllensten K How cognitive behavioural, rational emotive behavioural or multimodal coaching could prevent mental health problems, enhance performance and reduce work related stress. J Ration-Emot Cogn-Behav Ther (2008) 26:38–52. 10.1007/s10942-007-0069-y

[16] Gardner B Rose J Mason O Tyler P Cushway D Cognitive therapy and behavioural coping in the management of work-related stress: An intervention study. Work Stress (2005) 19:137–52. 10.1080/02678370500157346

[17] Alfonsson S Olsson E Hursti T The effects of therapist support and treatment presentation on the clinical outcomes of an Internet based applied relaxation program. Internet Interv (2015) 2:289–96. 10.1016/j.invent.2015.07.005

[18] Ebert DD Van Daele T Nordgreen T Karekla M Compare A Zarbo C Internet- and mobile-based psychological interventions: applications, efficacy, and potential for improving mental health. Eur Psychol (2018) 23:167–87. 10.1027/1016-9040/a000318

[19] Baumeister H Reichler L Munzinger M Lin J The impact of guidance on internet-based mental health interventions — a systematic review. Internet Interv (2014) 1:205–15. 10.1016/j.invent.2014.08.003

[20] Andersson G Cuijpers P Carlbring P Riper H Hedman E Guided internet-based vs. face-to-face cognitive behavior therapy for psychiatric and somatic disorders: a systematic review and meta-analysis. World Psychiatry Off J World Psychiatr Assoc WPA (2014) 13:288–95. 10.1002/wps.20151 PMC4219070 25273302

[21] Eisen KP Allen GJ Bollash M Pescatello LS Stress management in the workplace: A comparison of a computer-based and an in-person stress-management intervention. Comput Hum Behav (2008) 24:486–96. 10.1016/j.chb.2007.02.003

[22] Kuster AT Dalsbø TK Thanh BYL Agarwal A Durand-Moreau QV Kirkehei I Computer-based versus in-person interventions for preventing and reducing stress in workers. Cochrane Database Syst Rev (2017). 10.1002/14651858.CD011899.pub2 PMC6483691 28853146

[23] Wolever RQ Bobinet KJ McCabe K Mackenzie ER Fekete E Kusnick CA Effective and viable mind-body stress reduction in the workplace: a randomized controlled trial. J Occup Health Psychol (2012) 17:246–58. 10.1037/a0027278 22352291

[24] Andersson G Titov N Advantages and limitations of Internet-based interventions for common mental disorders. World Psychiatry (2014) 13:4–11. 10.1002/wps.20083 24497236 PMC3918007

[25] Barak A Hen L Boniel-Nissim M Shapira N A comprehensive review and a meta-analysis of the effectiveness of internet-based psychotherapeutic interventions. J Technol Hum Serv (2008) 26:109–60. 10.1080/15228830802094429

[26] Zetterqvist K Maanmies J Ström L Andersson G Randomized Controlled Trial of Internet-Based Stress Management. Cognit Behav Ther (2003) 32:151–60. 10.1080/16506070302316 16291546

[27] Ebert DD Lehr D Boß L Riper H Cuijpers P Andersson G Efficacy of an internet-based problem-solving training for teachers: results of a randomized controlled trial. Scand J Work Environ Health (2014) 40:582–96. 10.5271/sjweh.3449 25121986

[28] Hasson D Anderberg UM Theorell T Arnetz BB Psychophysiological effects of a web-based stress management system: a prospective, randomized controlled intervention study of IT and media workers [ISRCTN54254861]. BMC Public Health (2005) 5:78. 10.1186/1471-2458-5-78 16042796 PMC1183223

[29] Hintz S Frazier PA Meredith L Evaluating an online stress management intervention for college students. J Couns Psychol (2015) 62:137–47. 10.1037/cou0000014 24635586

[30] Ebert DD Berking M Thiart H Riper H Laferton JAC Cuijpers P Restoring depleted resources: Efficacy and mechanisms of change of an internet-based unguided recovery training for better sleep and psychological detachment from work. Health Psychol (2015) 34:1240–51. 10.1037/hea0000277 26651465

[31] Beatty L Lambert S A systematic review of internet-based self-help therapeutic interventions to improve distress and disease-control among adults with chronic health conditions. Clin Psychol Rev (2013) 33:609–22. 10.1016/j.cpr.2013.03.004 23603521

[32] Urech C Grossert A Alder J Scherer S Handschin B Kasenda B Web-based stress management for newly diagnosed patients with cancer (stream): a randomized, wait-list controlled intervention study. J Clin Oncol Off J Am Soc Clin Oncol (2018) 36:780–8. 10.1200/JCO.2017.74.8491 PMC5844668 29369731

[33] Heber E Ebert DD Lehr D Cuijpers P Berking M Nobis S The benefit of web- and computer-based interventions for stress: a systematic review and meta-analysis. J Med Internet Res (2017) 19:e32. 10.2196/jmir.5774 28213341 PMC5336602

[34] Domes G Stächele T von Dawans B Heinrichs M Effects of internet-based stress management on acute cortisol stress reactivity: Preliminary evidence using the Trier Social Stress Test for Groups (TSST-G). Psychoneuroendocrinology (2019) 105:117–22. 10.1016/j.psyneuen.2018.12.001 30573351

[35] Schiller H Söderström M Lekander M Rajaleid K Kecklund G A randomized controlled intervention of workplace-based group cognitive behavioral therapy for insomnia. Int Arch Occup Environ Health (2018) 91:413–24. 10.1007/s00420-018-1291-x PMC5908834 29387936

[36] Seyffert M Lagisetty P Landgraf J Chopra V Pfeiffer PN Conte ML Internet-delivered cognitive behavioral therapy to treat insomnia: a systematic review and meta-analysis. PloS One (2016) 11:e0149139. 10.1371/journal.pone.0149139 26867139 PMC4750912

[37] Ebert DD Heber E Berking M Riper H Cuijpers P Funk B Self-guided internet-based and mobile-based stress management for employees: results of a randomised controlled trial. Occup Environ Med (2016) 73:315–23. 10.1136/oemed-2015-103269 26884049

[38] Awa WL Plaumann M Walter U Burnout prevention: A review of intervention programs. Patient Educ Couns (2010) 78:184–90. 10.1016/j.pec.2009.04.008 19467822

[39] Ruwaard J Lange A Bouwman M Broeksteeg J Schrieken B. E-mailed standardized cognitive behavioural treatment of work-related stress: a randomized controlled trial. Cognit Behav Ther (2007) 36:179–92. 10.1080/16506070701381863 17852171

[40] Granath J Ingvarsson S von Thiele U Lundberg U Stress management: a randomized study of cognitive behavioural therapy and yoga. Cognit Behav Ther (2006) 35:3–10. 10.1080/16506070500401292 16500773

[41] Ahtinen A Mattila E Välkkynen P Kaipainen K Vanhala T Ermes M Mobile mental wellness training for stress management: feasibility and design implications based on a one-month field study. JMIR MHealth UHealth (2013) 1:e11. 10.2196/mhealth.2596 25100683 PMC4114468

[42] Ryan C Bergin M Chalder T Wells JS Web-based interventions for the management of stress in the workplace: Focus, form, and efficacy. J Occup Health (2017) 59:215–36. 10.1539/joh.16-0227-RA PMC5478505 28320977

[43] Lazarus R F S Stress, Appraisal, and Coping. New York: Springer (1984).

[44] de Jong GM Emmelkamp PMG Implementing a stress management training: Comparative trainer effectiveness. J Occup Health Psychol (2000) 5:309–20. 10.1037/1076-8998.5.2.309 10784292

[45] Cohen S Kamarck T Mermelstein R A global measure of perceived stress. J Health Soc Behav (1983) 24:385–96. 10.2307/2136404 6668417

[46] Klein EM Brähler E Dreier M Reinecke L Müller KW Schmutzer G The German version of the Perceived Stress Scale – psychometric characteristics in a representative German community sample. BMC Psychiatry (2016) 16:159. 10.1186/s12888-016-0875-9 27216151 PMC4877813

[47] Eskildsen A Fentz HN Andersen LP Pedersen AD Kristensen SB Andersen JH Perceived stress, disturbed sleep, and cognitive impairments in patients with work-related stress complaints: a longitudinal study. Stress Amst Neth (2017) 20:371–8. 10.1080/10253890.2017.1341484 28605986

[48] Hillhouse JJ Adler CM Walters DN A simple model of stress, burnout and symptomatology in medical residents: A longitudinal study. Psychol Health Med (2000) 5:63–73. 10.1080/135485000106016

[49] Antoni MH Lechner SC Kazi A Wimberly SR Sifre T Urcuyo KR How stress management improves quality of life after treatment for breast cancer. J Consult Clin Psychol (2006) 74:1143–52. 10.1037/0022-006X.74.6.1143 PMC5752106 17154743

[50] Wirtz PH Thomas L Domes G Penedo FJ Ehlert U Nussbeck FW Psychoendocrine validation of a short measure for assessment of perceived stress management skills in different non-clinical populations. Psychoneuroendocrinology (2013) 38:572–86. 10.1016/j.psyneuen.2012.07.017 22939272

[51] Glaesmer H Grande G Braehler E Roth M The german version of the Satisfaction With Life Scale (SWLS): Psychometric properties, validity, and population-based norms. Eur J Psychol Assess (2011) 27:127–32. 10.1027/1015-5759/a000058

[52] Hinz A Glaesmer H Brähler E Löffler M Engel C Enzenbach C Sleep quality in the general population: psychometric properties of the Pittsburgh Sleep Quality Index, derived from a German community sample of 9284 people. Sleep Med (2017) 30:57–63. 10.1016/j.sleep.2016.03.008 28215264

[53] Buysse DJ Reynolds CF Monk TH Berman SR Kupfer DJ The Pittsburgh sleep quality index: A new instrument for psychiatric practice and research. Psychiatry Res (1989) 28:193–213. 10.1016/0165-1781(89)90047-4 2748771

[54] Gerber M Hartmann T Brand S Holsboer-Trachsler E Pühse U The relationship between shift work, perceived stress, sleep and health in swiss police officers. J Crim Justice (2010) 38:1167–75. 10.1016/j.jcrimjus.2010.09.005

[55] Büssing A Perrar K-M Die Messung von Burnout. Untersuchung einer deutschen Fassung des Maslach Burnout Inventory (MBI-D). [Measuring burnout: A study of a German version of the Maslach Burnout Inventory (MBI-D).]. Diagnostica (1992) 38:328–53. 8004334

[56] Oncel S Ozer ZC Efe E Work-related stress, burnout and job satisfaction in turkish midwives. Soc Behav Pers Int J (2007) 35:317–28. 10.2224/sbp.2007.35.3.317

[57] Bellingrath S Weigl T Kudielka BM Chronic work stress and exhaustion is associated with higher allostastic load in female school teachers. Stress (2009) 12:37–48. 10.1080/10253890802042041 18951244

[58] Martin A Rief W Klaiberg A Braehler E Validity of the Brief Patient Health Questionnaire Mood Scale (PHQ-9) in the general population. Gen Hosp Psychiatry (2006) 28:71–7. 10.1016/j.genhosppsych.2005.07.003 16377369

[59] Knudsen HK Ducharme LJ Roman PM Job stress and poor sleep quality: Data from an American sample of full-time workers. Soc Sci Med (2007) 64:1997–2007. 10.1016/j.socscimed.2007.02.020 17363123 PMC1933584

[60] Smith ML Glass GV Miller TI The benefits of psychotherapy. Baltimore: Johns Hopkins University Press (1980).

[61] von Dawans B Kirschbaum C Heinrichs M The Trier Social Stress Test for Groups (TSST-G): a new research tool for controlled simultaneous social stress exposure in a group format. Psychoneuroendocrinology (2011) 36:514–22. 10.1016/j.psyneuen.2010.08.004 20843608

[62] Melville KM Casey LM Kavanagh DJ Dropout from internet-based treatment for psychological disorders. Br J Clin Psychol (2010) 49:455–71. 10.1348/014466509X472138 19799804

[63] Carlbring P Gunnarsdóttir M Hedensjö L Andersson G Ekselius L Furmark T Treatment of social phobia: randomised trial of internet-delivered cognitive-behavioural therapy with telephone support. Br J Psychiatry (2007) 190:123–8. 10.1192/bjp.bp.105.020107 17267928

